# Optogenetic Activation of a Lateral Hypothalamic-Ventral Tegmental Drive-Reward Pathway

**DOI:** 10.1371/journal.pone.0158885

**Published:** 2016-07-07

**Authors:** Eduardo D. Gigante, Faiza Benaliouad, Veronica Zamora-Olivencia, Roy A. Wise

**Affiliations:** Intramural Research Program, National Institute on Drug Abuse, National Institutes of Health, Department of Health and Human Services, Baltimore, Maryland, United States of America; University of Chicago, UNITED STATES

## Abstract

Electrical stimulation of the lateral hypothalamus can motivate feeding or can serve as a reward in its own right. It remains unclear whether the same or independent but anatomically overlapping circuitries mediate the two effects. Electrical stimulation findings implicate medial forebrain bundle (MFB) fibers of passage in both effects, and optogenetic studies confirm a contribution from fibers originating in the lateral hypothalamic area and projecting to or through the ventral tegmental area. Here we report that optogenetic activation of ventral tegmental fibers from cells of origin in more anterior or posterior portions of the MFB failed to induce either reward or feeding. The feeding and reward induced by optogenetic activation of fibers from the lateral hypothalamic cells of origin were influenced similarly by variations in stimulation pulse width and pulse frequency, consistent with the hypothesis of a common substrate for the two effects. There were, however, several cases where feeding but not self-stimulation or self-stimulation but not feeding were induced, consistent with the hypothesis that distinct but anatomically overlapping systems mediate the two effects. Thus while optogenetic stimulation provides a more selective tool for characterizing the mechanisms of stimulation-induced feeding and reward, it does not yet resolve the question of common or independent substrates.

## Introduction

Electrical stimulation of the same sites in the lateral hypothalamic area (LHA) can cause a drive-like state—inducing feeding in sated animals—or it can serve as a reward in its own right [1,2]. That rats are willing to work for stimulation that produces a hunger-like drive state [3] is counter-intuitive and is known as the “drive-reward paradox” [4]. Evidence that that the two effects are mediated by anatomically overlapping but functional independent systems would resolve the paradox, but attempts to dissociate the mechanisms of the two stimulation affects have been largely unsuccessful. For example, the two effects are mediated by neuronal populations with the same ranges of refractory periods and conduction velocities, and with the same fiber alignment between the LHA and the ventral tegmental area (VTA) [5,6]. Recent optogenetic studies suggest that each effect is mediated by a GABAergic and not a glutamatergic projection to the VTA from lateral hypothalamic cells of origin [7–10]. While feeding and self-stimulation are differentially influenced by high and low frequency optogenetic stimulation under the conditions of one such study [10], this has not been confirmed under reasonably similar conditions in another [7].

The LHA was initially identified as a feeding center by some workers [11,12], and as a pleasure center by others [13,14]. The suggestion in each case was that the cells of this region received and integrated specialized inputs related to the function in question. However, stimulation in this and other segments of the medial forebrain bundle (MFB) can also induce a variety of other biologically primitive, species-typical, behaviors (e.g., drinking, predatory attack, copulation, gnawing, nest building [15]), raising the possibility that the system serves some form of general arousal function [16–18] that is relevant to both the instigation of actions that follow the stimulation and to the reinforcement of acts that preceded it [4].

The intrinsic neurons of the LHA form a segment the interstitial or “bed” nuclei feeding efferent fibers into the (MFB) [19,20]. Several electrical stimulation studies implicate MFB fibers of passage from more rostral or caudal regions (as well as fibers from the LHA itself) in the behavioral effects of electrical stimulation. Electrical stimulation activates fibers of passage more readily than cells of origin [21], and feeding deficits similar to those caused by LHA lesions are caused by selective degeneration of ascending dopaminergic fibers that pass through or near the LHA before turning to more rostrolateral targets [22,23]. Moreover, electrical stimulation of the MFB at sites rostral or caudal to the LHA can be rewarding [24–27]. Electrical stimulation of the MFB caudal to the LHA, like LHA stimulation itself, can induce feeding [5,28]. Thus electrical stimulation studies cannot confirm whether the feeding or reward caused by lateral hypothalamic stimulation results from activation of fibers that originate from the LHA proper.

Parametric electrical stimulation studies have confirmed that fibers linking the LH and VTA are involved in both the rewarding [29] and the feeding [6] effects of stimulation at each site. The distributions of refractory periods for the fibers mediating the two effects are much the same [5,29,30], and collision tests implicate connectivity of activated fibers between these two sites for both effects [6,29,30]. The direction of propagation of reward signals is primarily from rostral to caudal [31], confirming earlier evidence that the major portion of the directly activated substrate (the “first stage” neurons with respect to LHA stimulation) does not involve the high-threshold dopamine fibers [32] that ascend from the VTA through the LHA to limbic and cortical targets [33]. These studies do not reveal, however, whether the fibers in question originate from cells in, let alone restricted to, the LHA.

Optogenetic stimulation offers a way to selectively activate fibers originating from more or less localized cell populations [34]. Such studies in mice have confirmed that selective activation of GABAergic fibers originating in the bed nucleus of LHA and projecting to the VTA can be rewarding, can induce sated mice to eat dry lab chow, and can increase the ingestion and seeking of highly palatable sucrose solutions [7,8,10,35]. In the present study we used optogenetic stimulation to address the question of whether cells localized to the LHA are uniquely involved in feeding and reward or whether more rostral and caudal cells also contribute. Electrical stimulation studies show that activation of fibers connecting the lateral preoptic area (LPO) with the LHA [36] and fibers connecting the LHA with the VTA [29] but not necessarily fibers connecting the LPO with the VTA [36] is rewarding. Optogenetic stimulation findings of Kempadoo et al. [35] suggest (but do not confirm) contributions to reward function of a glutamatergic-neurotensinergic projection from the anterior hypothalamic area (AHA) to the VTA. Neurotensin afferents to the ventral tegmental area are most strongly expressed in the LPO and, to a lesser extent, the AHA [37], Glutamatergic efferents arise from bed nuclei at all hypothalamic levels of the medial forebrain bundle [38,39]. Thus our aim was to determine if selective optogenetic activation of axons projecting to the VTA from the LPO, AHA or posterior hypothalamic area (PHA) can cause reward or feeding similar to reward and feeding caused by activation of projections from the LHA proper. In addition, we addressed the question of whether the fibers mediating the two stimulation effects are similarly sensitive to variations in pulse width, pulse frequency, and train length.

## Materials and Methods

### Animals

Male Sprague-Dawley rats (Charles Rivers USA, Baltimore) weighing 275-350g were used. They were individually housed in a temperature and humidity-controlled room with a reverse 12-h dark-light cycle (lights off at 0800h) with free access to dry food and water. Animal procedures were performed in accordance to the Guide for the Care and Use of Laboratory Animals published by the National Institutes of Health and were approved by the Animal Care and Use Committee of the National Institute on Drug Abuse Intramural Research Program.

### Surgery

Under isoflurane anesthesia (2–3% isoflurane in 1L/min O_2_), each rat was injected with a viral vector targeting one of the medial forebrain bundle sites of interest and a fiber optics probe just dorsal to the ventral tegmental area. The tissue over the skull was resected, and subcutaneous injections of the local anesthetic Marcain (0.1 ml of a 0.5% solution) were given at the wound margins. Expression of the light sensitive cation channel channelrhodopsin-2 (CHr2) and the marker enhanced yellow flourescent protein) were induced by unilateral injection of 0.5μl the viral construct rAAV5-CamKII-hChR2(H134R)-eYFP (University of North Carolina Vector Core) at a rate of 0.1μl/min. The four injection targets, separated by 1.0 mm in the anterior-posterior plane, were the lateral preoptic area (LPO: AP -0.8), anterior hypothalamic area (AHA: AP -1.8), lateral hypothalamic area (LHA: AP -2.8), and posterior hypothalamic area (PHA: AP-3.8); the medial lateral (ML 1.7) and dorsal ventral (DV -8.4) coordinates were common to the four sites (coordinates according to [40]). A group of 8 animas was given viral injections at the lateral hypothalamic coordinates containing the construct for eYFP but lacking the construct for ChR2. For each animal, a 200μm fiber optics probe (Precision Fiber Products) was lowered to a target just dorsal to the VTA. Coordinates were 7.9mm from the surface of the skull at an angle of 10° toward the midline from an entry point 5.4mm posterior to bregma and 1.5mm lateral to the midline ipsilateral to the viral injection. The probe was anchored to stainless steel skull screws with dental acrylic. Banamaine (2 mg, s.c.) and saline (3ml, s.c.) were given at the end of surgery. Five weeks were allowed for recovery prior and expression of ChR2 before the start of behavioral testing.

### Apparatus

Each rat was tested for stimulation-induced feeding and self-stimulation under dim red illumination in a standard operant chamber (Med Associates) equipped with two nose-poke ports with white cue lights above the ports. During testing the rats were connected to a 200μm fiber optic cable (Thorlabs) through a rotary optic joint (Doric Lenses) to a laser (OEM Laser Systems) used to deliver 473nm blue light under the control of a digital pulse simulator (Master-9, AMP Instruments).

### Stimulation-induced feeding

Each animal was tested for stimulation-induced feeding and for self-stimulation; half the animals were tested for feeding first and half for self-stimulation first. Initial screening for stimulation-induced feeding involved 10 one-minute trials with continuous 20Hz trains of 5msec laser pulses interspersed with 10 one-minute unstimulated comparison trials. Approximately 40g of dry lab chow was scattered on the floor of the test chamber and latency to pick up and eat food was scored in each stimulation or no-stimulation trial. Eating was identified as stimulation-induced if three criteria were met: (i) the animal initiated eating in 8 of the 10 daily stimulation periods on each of 5 consecutive days of testing, (ii) once eating was initiated within a trial it continued until the stimulation terminated at the end of the trial, and (iii) eating was never initiated in the one-minute no-stimulation trials. Latencies to first bite and amounts eaten were each recorded.

Twelve rats that both ate and self-stimulated in response to 20Hz stimulation were tested further under several variations in stimulation parameters. Four stimulation frequencies (10, 20, 40, and 80Hz) were each tested with each of three pulse widths (2.5, 5.0, and 10msec); stimulation parameters were tested on separate days and given in a counterbalanced sequence. The 8 animals infected with eYFP without ChR2 were tested with this group and in the parametric testing for self-stimulation.

### Self-stimulation

Each rat was independently trained and tested in a nose-poke apparatus to determine if optical stimulation was rewarding. In initial screening, each interruption of a photocell beam in one of two ports (the “active” port) triggered a 1.5sec train of 5ms pulses of optical stimulation at 20 Hz. Each nose poke also activated a white cue light above the active port. Responding during a stimulation train or in the other (“inactive”) port was without scheduled consequence. Rats were trained in daily 10-min sessions until the rate of beam interruptions varied less than 10% over 3 consecutive days. Animals that failed to perform at least 20 active nose-poke responses per session were considered as non-responders and were not used further. Rats that displayed stable response rates were tested in a reversal task in which nose-poking in the previously active port was no longer effective but poking in the previously inactive port became effective.

The twelve rats that were tested for feeding under parametric variations were tested for self-stimulation with the same stimulation frequencies (10, 20, 40, and 80Hz) and the same three pulse widths (2.5, 5.0, and 10msec) and with each of six train durations (0.2, 0.4, 0.8, 1.6, 3.2, and 6.4 sec); each combination was tested on a separate day and the combinations were tested in counterbalanced sequence.

### Histology

Following completion of testing the rats were anesthetized with equithesin (3 ml/kg, i.p.) or isoflurane and were transcardically perfused with a phosphate buffer solution (3.85 g of NaOH and 16.83 g of NaH_2_PO_4_ in 1L of distilled water) following by a 4% paraformaldehyde solution. Brains were removed, left in 4% paraformaldehyde for 2 hours at 4°C and dehydrated overnight in a phosphate buffer solution containing 18% sucrose at 4°C. The next day, the brains were transferred to a fresh phosphate buffer solution containing 18% sucrose at 4°C for 3 hours before being frozen and stored at -80°C. Sagittal or coronal sections were subsequently taken at 20 um and stored at -80°C in 0.1% DEPC-treated water solution followed by 30% sucrose, 30% ethylene glycol and 2mM of NaN3.

Brain sections were processed for eYFP-immunodetection as follows: free-floating coronal sections were incubated for 1 h in PB supplemented with 4% bovine serum albumin and 0.3% Triton X-100. Sections were then incubated with mouse anti-GFP antibody (1:1000, 632381, Clontech Laboratories, Mountain View, CA) overnight at 4°C. After rinsing 3 × 10 min in PB and incubated in biotinylated goat anti-mouse antibody (1:200, Vector Laboratories, Burlingame, CA); then, sections were rinsed with PB and incubated for 1 h at room temperature in avidin-biotinylated horseradish peroxidase (1:200, ABC kit, Vector Laboratories). Sections were rinsed, and the peroxidase reaction was developed with 0.05% 3,3’-diaminobenzidine and 0.03% H_2_O_2_. Sections were mounted on coated slides. Bright field images were collected with an Olympus MVX10 with 0.63 objective (Olympus, Waltham, MA).

### Statistics

The effects of pulse width and pulse frequency were assessed in the feeding study with 2-way and in the self-stimulation study with 3-way within-subjects analysis of variance (ANOVA). Post hoc comparisons were made using Bonferroni correction. When the assumption of sphericity was not met, conservative Greenhouse-Geisser corrections were made to the degrees of freedom. Separate within-subjects analyses were done for train lengths of 200-800msec and 1600-6400msec because independent groups were used for the two ranges. Linear and quadratic trends were assessed with 1-way ANOVAs for each frequency in the feeding study. Calculations were performed using SPSS software (IBM Corporation).

## Results

Optical stimulation of the VTA induced feeding and was rewarding in some but not all animals with viral transfection of ChR2 plus eYFP into cells of origin at various levels of the hypothalamic MFB. The effectiveness of stimulation varied with the site of the viral injections, as discussed in the following section. Optical stimulation of the VTA never induced feeding and was never rewarding in any animal transfected with eYFP but not ChR2.

### Localization

**Fig 1:**
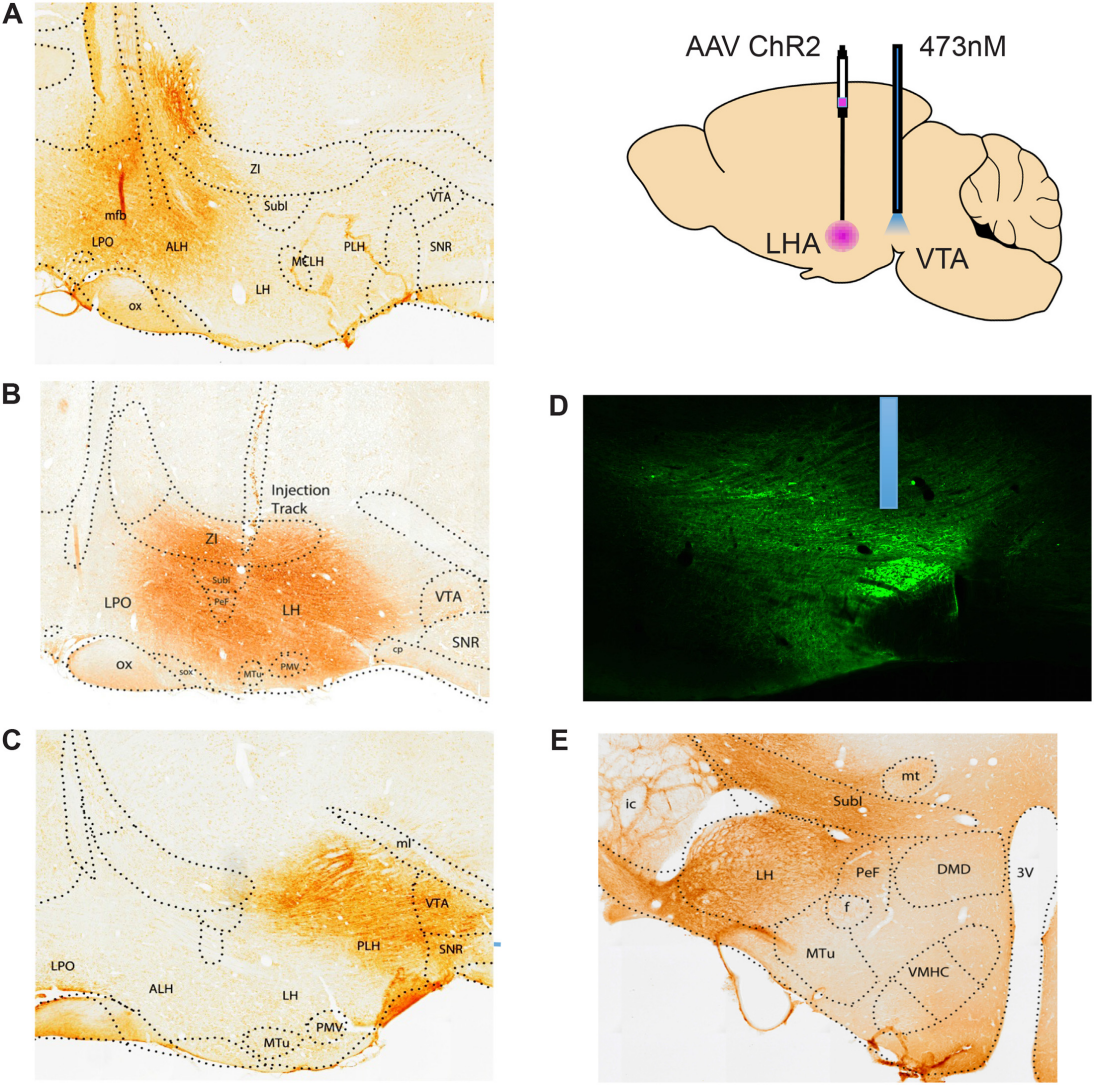
Localization of viral infections and optic probes. A. Sagittal section showing the infected region in an animal with an AHA viral injection. B. An LHA infection. C. A PHA infection. D. Fluorescence photomicrograph showing the location of a probe just dorsal the fluorescent accumulation in fibers infected by an LHA viral injection. E. A coronal section showing the size of the viral infection. Viral injections for the non-responsive LPO animals, not shown, were 1mm rostral to the AHA site.

Viral infections spread approximately 1mm from each injection site; thus the infections at each site overlapped considerably with those of neighboring sites (Fig 1A–1C). Optic probes terminated just dorsal to the fluorescent fibers that converged in the VTA (Fig 1D). A total of 84 animals were screened for stimulation-induced feeding and for self-stimulation. Twenty-one of the animals with LHA viral injections and two with AHA injections both ate and self-stimulated; nine animals with LHA, one with PHA and one with AHA injections ate but did not self-stimulate; and two AHA, one LHA, and four PHA animals self-stimulated but did not eat (Table 1). No eating or self-stimulation was obtained from any animal with a viral injection in lateral preoptic area. Three of the animals with PHA injections that self-stimulated but did not eat showed aversive responses to stimulation; they ran away from (but returned to) the nose-poke receptacle for short trains of stimulation and they hyperlocomoted, occasionally picking up food but not staying put to eat it, during the long trains of stimulation in the feeding test.

**Table 1:** Numbers of cases in each group responding to optogenetic stimulation with feeding (Eat) or self-stimulation (SS).

Behavior	LPO	AHA	LHA	PHA
Eat plus SS	0	2	21	0
Eat only	0	1	9	1
SS only	0	2	1	4
Neither	9	12	10	12
Totals	9	17	41	17

### Parametric comparisons

**Fig 2:**
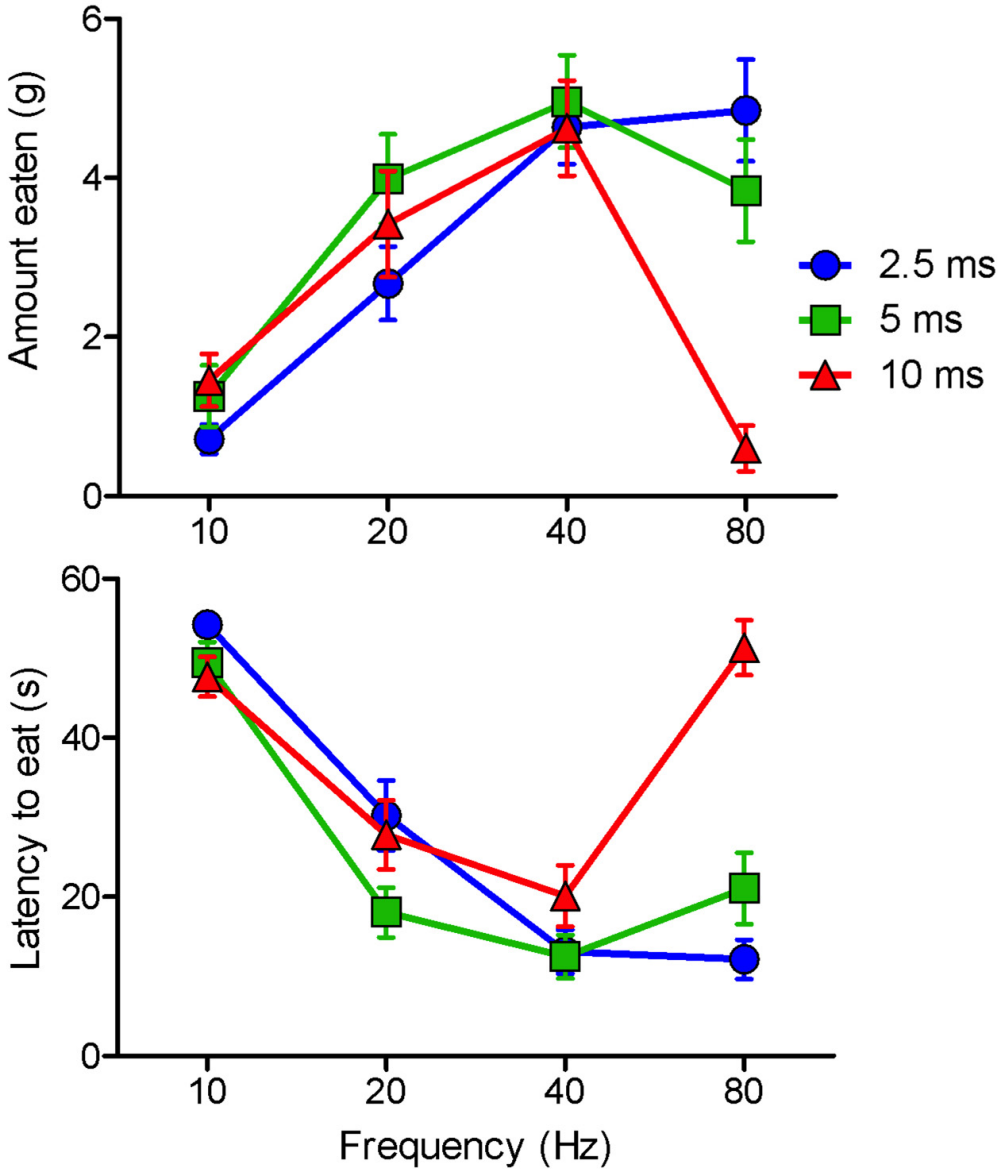
Amounts eaten and latencies to feed (means and standard errors) as a function of pulse width and pulse frequency in response to optical stimulation of VTA fibers originating from cells of the bed nucleus of the LHA. Note that stimulation at 80Hz is almost continuous when 10msec pulses are given.

**Fig 3:**
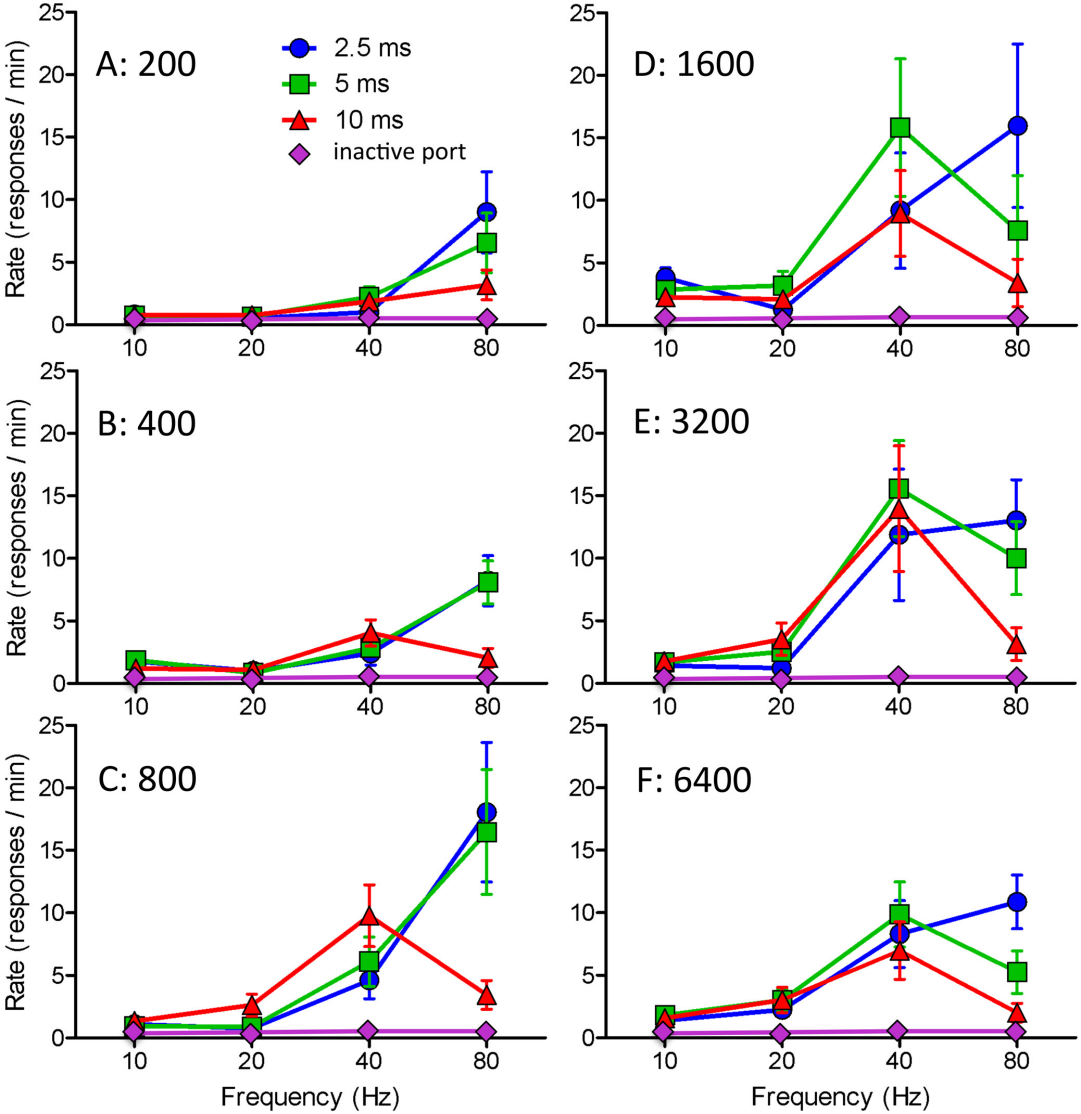
Rate of nosepoking as a function of pulse width, pulse frequency, and train length (means and standard errors) for optical stimulation of VTA fibers originating from cells of the bed nucleus of the LHA.

The effectiveness of stimulation varied as complex functions of pulse width and pulse frequency in the case of feeding (Fig 2) and of pulse width, pulse frequency, and train length in the case of self-stimulation (Fig 3). Despite significant main effects of Pulse Width and Pules Frequency for feeding latency (F_2,24_ = 10.447, *P*<0.001 and F_1.661,19.934_ = 49.936, *P*<0.001), amounts eaten (F_2,24_ = 33.357, *P*<0.001 and F_3,36_ = 91.982, *P*<0.001), and self-stimulation rate (short trains: F_1.32,14.52_ = 5.401, *P*<0.03 and F_1.065,11.72_ = 11.654, *P*<0.005; long trains: F_2,16_ = 7.251, *P*<0.006 and F_1.01,8.08_ = 8.646, *P*<0.02), there were in each case significant Pulse Width X Pulse Frequency interactions (eating latency: F_1.805,19.856_ = 7.385, *P*<0.005; amount eaten: F_2.728,32.731_ = 20.355, *P*<0.001; response rate [short trains]: F_1.805,19.856_ = 7.385, *P*<0.005; response rate [long trains] F_2.449,19.594_ = 13.099, *P*<0.001). The interactions reflected the fact that whereas the effects of stimulation frequency were predominantly linear in the case of short pulse duration, they were markedly non-linear in the case long pulse duration. This is illustrated in the case of amounts eaten by a strong linear and a weak quadratic trend for the 2.5ms pulse width (F_1,36_ = 111.57, *P*<0.0001 and F_1,36_ = 8.23, *P*<0.007, respectively) and a strong quadratic trend and an insignificant linear trend (F_1,36_ = 84.91, *P*<0.0001 and F_1,36_ = 0.88, *P* = 0.36, respectively) for the 10m pulse width (comparison of the F values, given equal degrees of freedom, gives a better quantitative comparison than comparison of the *P* values.). In the case of each of the eight testing conditions, the 2.5ms pulses were more effective at 80Hz than at 40Hz (binomial theorem: *P*<0.02) whereas in each of the eight conditions the 10ms pulses were less effective at 80Hz than at 40Hz (*P*<0.02). In each case 5m pulses at 80Hz was intermediate to the strong effect of 2.5ms pulses and the weak effect of 80Hz pulses (*P*<0.002 on the hypothesis that the probability of an intermediate value on any one trial is <0.34).

The most effective frequency for stimulation-induced eating was 80Hz with 2.5ms pulses and 40Hz with 5 or 10ms pulses. The most effective frequency for self-stimulation was 80Hz for 2.5ms pulses, 80Hz for short trains and 40Hz for long trains of 5ms pulses, 40Hz for long trains and all but the shortest train of 10ms pulses (Fig 3). Amounts eaten were negatively correlated with latency to start eating; correlation coefficients for the 2.5, 5, and 10ms conditions ranged from 0.995 to 0.993 (*P*<0.001 in each case).

### Other behaviors

Animals with LHA viral injections that ate in response to 20Hz stimulation sometimes picked up cardboard pieces when tested with food and cardboard present; on each such occasion, however, they simply moved the cardboard aside and picked up and began eating a food pellet. In the nine animals that ate in response to stimulation and were subsequently tested with water rather than food, no instances of stimulation-induced drinking were seen at any of the combinations of parameters previously used in the feeding tests.

## Discussion

At the stimulation parameters used—and as it the case with electrical stimulation [3]—the feeding resembled that of a hungry animal and was sensitive to the feedback from the 4-5g of food eaten within sessions at optimal parameters. There were no signs of circling or compulsive gnawing or licking at the floor of the cage, as has been seen under different testing conditions in some mice [7] but not others [10]. Self-stimulation was slower than is typically seen with electrical stimulation of the MFB, but this was expected from the fact that electrical stimulation opens native voltage-gated cation channels much more immediately than light opens transfected light-gated channels [41], and was consistent with optogenetic reports involving stimulation of the same pathway in mice [10].

The present findings suggest a substrate for both feeding and reward that originates in cells of the lateral hypothalamic area at the anterior-posterior level of the ventromedial nucleus—the area classically associated with feeding—projects to or through the ventral tegmental area and does not involve fibers from more rostral or caudal bed nuclei of the MFB. The fact that the anterior-posterior level of the lateral hypothalamus is identified with respect to the adjacent ventromedial nucleus reflects the fact that the lateral hypothalamic area is merely a portion of ascending columns of glutamatergic and GABAergic neurons that form bed nuclei of the medial forebrain bundle. There are no clear boundaries of this region with respect to the cells infected by our viral injections, and the considerable overlap of our injections does not provide such boundaries. Nonetheless, neither eating nor self-stimulation was seen in cases that were not in or adjacent to the LHA, and only fragmentary findings were seen in cases not targeting the LHA. The findings, while consistent with the view that a common substrate mediates the two findings, do not falsify the alternative and do not resolve the question of common or separate substrates. Whereas the region cannot be delimited by the boundaries of glutamatergic or GABAergic nuclei, several more punctate cell groups can be identified by co-expressed neuropeptides. It is possible that the targeting of ChR2 to selected neuropeptide-expressing populations will allow a precise delimitation of the critical substrates for feeding and reward in this region; such studies will offer further opportunities to dissociated the mechanisms of the two behaviors.

A common system is suggested by the findings that both responses were seen in the majority of animals that showed either response but a common system is questioned by the findings that 11 animals ate but did not self-stimulate and seven that self-stimulated but did not eat. All the animals that ate or self-stimulated were in the lateral hypothalamic group or one of the adjacent groups with viral infections that overlapped the lateral hypothalamus. If the eaters that did not self-stimulate had all been from one group and the self-stimulators that did not eat from another group, this would have been strong evidence for separate but overlapping systems. This, however, was not the case. Animals that ate but did not self-stimulate were distributed across the AHA, LHA, and PHA groups, and animals that self-stimulated but did not eat were distributed across both the AHA and PHA groups. Our working hypothesis is that the behaviors in the AHA and PHA animals were less reliable simply because their infections only partially invaded the critical lateral hypothalamic substrate or substrates. None of the lateral preoptic animals, whose infections overlapped those of the anterior but not the lateral hypothalamic groups showed any inclination to eat or self-stimulate.

It is difficult to understand the finding that nine of the 11 animals that ate but did not self-stimulate were within the group with LHA viral injections. Given the size of the infections, it is difficult to imagine that small differences in the stereotaxic accuracy of the viral injections could account for the lack of self-stimulation in these animals; small differences in electrode placement do not cause significant differences in feeding or self-stimulation induced by electrical stimulation [42,43]. Small differences in optic probe placement are also unlikely to be a factor; feeding and reward have been obtained with varying probe placements in the ventral tegmental area in the mouse, some [10] similar to the present placement and some [7] considerably more lateral. Thus there is no obvious explanation for why 21 LHA animals both ate and self-stimulated and nine ate but did not self-stimulate. Nor is there any obvious explanation for why 11 of 41 animals with LHA viral injections neither ate nor self-stimulated. It is important to note, however, that a significant number of animals also fail to eat in response to lateral hypothalamic electrical stimulation [11,16], and that the failures cannot be explained by differences in stimulation locus [42,44].

The parametric findings are consistent with findings from electrical stimulation studies [5,6] suggesting further similarities between stimulation induced feeding and reward. Their directly activated mechanisms have similar refractory periods, conduction velocities, and alignment of lateral hypothalamic to ventral tegmental fibers. In the present study, stimulation was progressively more effective for both responses over the range of 10 to 40Hz and was even more effective, again for both responses, at 80Hz when 2.5msec (but not 5 or 10msec) pulses were used. This appears not to be a universal finding; while one recent mouse study involving optogenetic stimulation of GABAergic fibers from LHA to VTA indicated that higher frequencies were most effective for stimulation-induced feeding [7], a similar study from our lab feeding indicated that low frequency (5Hz) stimulation was optimal [10]. Further study is needed to identify the critical differences between these studies; one difference between the mouse studies was the lateral coordinate for the optical probe. The strong conclusion to be drawn from our parametric data is that there are interactions between pulse width, pulse frequency, and train length. It will be surprising if there are not further interactions with laser power; increasing laser power not only enlarges the illuminated field; it can also increase the frequency of evoked action potentials [41].

The viral approach used in the present study established non-selective optogenetic control of both GABAergic and glutamatergic projections from the LHA to the VTA [7]. It has been suggested that rewarding effects of optogenetic stimulation of VTA terminals in this preparation might result from activation of the glutamatergic projection [35], but this hypothesis does not appear to hold for feeding; selective activation of the GABAergic projection from LHA to VTA induces feeding and is rewarding [7,10], but selective activation of the glutamatergic projection does not induce feeding or approach to food-associated stimuli [7].

As expected from electrical stimulation studies, response rates in the present study were inversely related to train duration. In large part, this reflects the fact that longer stimulation trains take up a greater portion of the test session and require fewer responses to maintain the same rate of stimulation. Another possible factor, however, is that stimulation that is initially rewarding often becomes aversive [45,46] or at least less rewarding [47] over time. This offers a potential solution to the drive-reward paradox; perhaps stimulation only induces feeding after it has started to become aversive. This suggestion is consistent with the finding that the preferred train lengths of electrical stimulation tend to be shorter than approximately 1.6 seconds, whereas the minimum train length for inducing feeding is about that same length [48]. In the present study, the shortest latencies at the optimal parameters for feeding (5msec pulses at 40Hz) were approximately 5 seconds, a bit less than the longest tested stimulation trains for self-stimulation. Thus, the present study leaves unresolved the question of whether the same or different substrates mediate feeding and reward function.

While the finding that activation of GABAergic but not glutamatergic fibers from the LHA to the VTA support feeding and self-stimulation suggests a common GABAergic substrate for the two behaviors, it does not rule out the possibility that different subsets of GABAergic fibers are responsible for the two effects. Calcium imaging and electrophysiological recording studies indicate subpopulations of LHA neurons, at least some of which project to VTA, that are involved in different aspects of feeding and food-approach. Thus it remains to be determined whether the same population of GABAergic LHA to VTA neurons mediates stimulation-induced eating and self-stimulation.

## Supporting Information

S1 File Manuscript Database. (XLSX)
